# Changes of intestinal microbiota and liver metabolomics in yellow catfish (*Pelteobagrus fulvidraco*) before and after rice flowering in rice-fish symbiosis farmed mode

**DOI:** 10.3389/fmicb.2025.1617168

**Published:** 2025-08-06

**Authors:** Rui Cheng, Zhuoqi Ying, Yang Yang, Chongying Zhang, Wei Zhou, Zhiming Zhang, Huiping Ding, Ya Zhou, Chi Zhang

**Affiliations:** ^1^Key Laboratory of Ecological Impacts of Hydraulic-Projects and Restoration of Aquatic Ecosystem of Ministry of Water Resources, Institute of Hydroecology, MWR &CAS, Wuhan, China; ^2^Hubei Key Laboratory of Animal Nutrition and Feed Science, School of Animal Science and Nutritional Engineering, Wuhan Polytechnic University, Wuhan, China; ^3^Chongqing Three Gorges Vocational College, Chongqing, China; ^4^Agricultural Technology and Machinery Extension Center of Wanzhou District, Chongqing, China

**Keywords:** 16S rRNA, intestinal microbiota, liver metabolomics, rice-fish symbiosis, yellow catfish

## Abstract

The rice-fish symbiosis farming model (RFFM) has been shown to enhance gut microbial diversity and improve immunity in fish. To examine changes in gut microbiota and hepatic metabolism in yellow catfish (*Pelteobagrus fulvidraco*) during different rice growth stages, we analyzed samples collected from the pre-flowering (Group P) and after-flowering (Group A) phases. Gut microbiota composition was assessed using 16S rRNA sequencing, with data analyzed using Principal component analysis (PCA), while hepatic metabolic profiles were characterized through untargeted metabolomics using XCMS and metaX for data processing. Our results revealed a significant increase in gut microbial diversity in Group A. Notably, the relative abundances of *Pseudomonas* and *Cetobacterium* were significantly lower in Group A compared to Group P, whereas Brevundimonas, *Oxyphotobacteria_unclassified*, and *Clostridium_sensu_stricto_1* were more abundant in Group A. Hepatic metabolic profiles also differed between the two groups, with amino acid metabolism and related pathways being upregulated, while lipid metabolism and associated pathways were downregulated in Group A. Correlation analysis using SPSS suggested that *Clostridium_sensu_stricto_1*, a dominant bacterial group, played a key role in mediating hepatic metabolic changes under the RFFM. These findings indicate that rice flowering in the rice-fish symbiosis system positively influences gut microbiota composition and hepatic metabolism in yellow catfish. Furthermore, *Clostridium_sensu_stricto_1* may have potential as a probiotic for improving fish health in this integrated farming system.

## 1 Introduction

The integrated rice-fish symbiosis farming model (RFFM) is an ecological agricultural system that combines rice cultivation with aquaculture, offering multiple benefits such as improved resource utilization, increased farmer income, and enhanced ecosystem stability (Sathoria and Roy, 2022). Yellow catfish (*Pelteobagrus fulvidraco*), an omnivorous species valued for its delicate flesh, is widely cultured in Asia (Kim and Lee, 2005). The RFFM, a specialized form of rice-aquatic farming, has been extensively implemented in China, Bangladesh, and India (Zhang et al., 2022). In this system, yellow catfish fry is reared in rice paddies during the rice growth period, where they prey on pests while their excrement serves as a natural fertilizer. This process reduces the need for pesticides and chemical fertilizers, promoting an ecologically sustainable planting-breeding model (Kaewpuangdee et al., 2024).

Recent studies highlight the critical role of gut microbiota in fish nutrition, metabolism, immunity, and environmental adaptation. For example, dietary supplementation with microencapsulated *Saccharomyces cerevisiae* enhances growth performance, feed conversion, and immune function in striped catfish (Boonanuntanasarn et al., 2019). Similarly, *Lactobacillus acidophilus* supplementation increases immune markers such as TGF-*β* (Transforming Growth Factor-β), IL-8 (Interleukin-8), and TNF-*α* (Tumor Necrosis Factor-α) in juvenile carp (Adeshina et al., 2020). Probiotic bacteria colonizing the fish gut also produce digestive enzymes that aid intestinal digestion (El-Haroun et al., 2006). However, most current aquatic probiotics originate from non-host species, leading to poor adaptability and low colonization efficiency (Zhang et al., 2025). Therefore, the development of fish-derived probiotics is essential for sustainable aquaculture. The liver, a key metabolic organ in fish, plays a crucial role in nutrient processing and immune regulation (Feng et al., 2025). Hepatic metabolism can be influenced by dietary composition, particularly variations in fat, protein, and carbohydrate intake (Chen-Liaw et al., 2025; Nguyen et al., 2025; Li et al., 2020) In the rice-fish symbiosis farming system, the availability of natural prey for yellow catfish increases following rice flowering. Gut microbiota composition plays a critical role in host metabolism, immunity, and environmental adaptation (Wang et al., 2024; Spiljar et al., 2017; Hernández-Chirlaque et al., 2016), with these regulatory effects often linked to dominant microbial populations (Singh et al., 2025). Moreover, the gut-liver axis plays a crucial role in fish metabolism and immune function. Specific bacterial species from *Bacteroidetes* and *Firmicutes* ferment dietary fibers to produce short-chain fatty acids (SCFAs), such as Clostridium_sensu_stricto_1, including acetate, propionate, and butyrate, which regulate hepatic metabolism and immune responses (Venegas et al., 2019). The RFFM has been shown to enhance gut microbial diversity (Venegas et al., 2019; Zhu et al., 2024) and improve immune function in fish (Samaddar et al., 2025; Liu et al., 2024). Additionally, after-flowering rice plants influence gut microbiota composition and hepatic metabolism in tilapia (Wang et al., 2022). However, it remains unclear whether similar effects occur in yellow catfish under the RFFM.

This study investigates the impact of rice flowering on gut microbiota and hepatic metabolism in yellow catfish. Using 16S rRNA sequencing and untargeted metabolomics, we compare gut microbial composition and hepatic metabolic profiles before and after rice flowering. The rice flowering period lasts approximately 15–20 days (Sun et al., 2022), during which abundant pollen, primarily from the anther wall, falls into the rice paddy. This pollen serves as a direct food source for omnivorous yellow catfish while also promoting the proliferation of natural prey, such as zooplankton and algae (Welde et al., 2024). Rice pollen is rich in amino acids, lipids, and carbohydrates, along with bioactive components like pectin, cellulose, and hemicellulose (Zhang et al., 2024). Previous studies have shown that pollen consumption can enhance gut microbial diversity in animals (El-Asely et al., 2014). Given these observations, we hypothesized that gut microbiota composition in yellow catfish differs between the pre-flowering (Group P) and after-flowering (Group A) stages. Additionally, we explore the interactions between key gut microbes and metabolites, providing a foundation for the potential development of yellow catfish-derived probiotics.

## 2 Materials and methods

### 2.1 Experimental design and sampling

**Figure 1:**
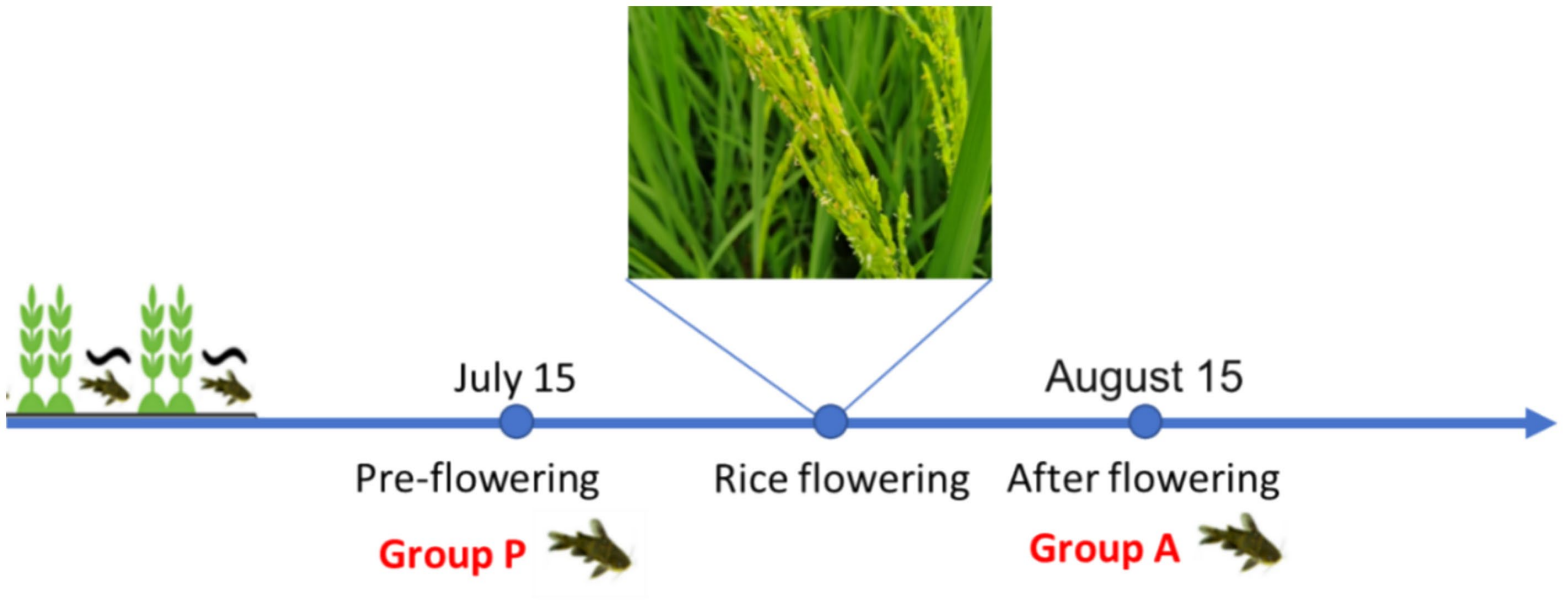
Schematic diagram of the rice-fish symbiosis farmed mode.

The experiment was conducted at a rice-fish symbiosis farm in Liangping Chongqing, China. Fish ditch were constructed, occupying 10% of the total paddy field area. Yellow catfish fry, with an initial body weight of 0.63 ± 0.05 g/tail (2 months of age), were stocked at a density of 1,000 individuals per 667m^2^. Rice seedlings were transplanted in late April 27 2020. On July 15, six yellow catfish (Weight of 9.66 ± 1.63 g/tail) were randomly sampled from the paddy and designated as the pre-flowering group (Group P). Intestinal contents and liver tissues were collected and immediately stored at −80°C for further analysis. A second sampling was conducted on August 15, when six additional yellow catfish(Weight of 12.48 ± 2.78 g/tail) were randomly selected and assigned to the after-flowering group (Group A) (Figure 1).

### 2.2 Gut microbiota analysis

#### 2.2.1 16S rRNA sequencing

Total DNA was extracted from the samples using the E. Z. N. A.® Stool DNA Kit (D4015, Omega Bio-tek, United States) following the manufacturer’s protocol. Nuclease-free water was used as a negative control to prevent contamination. The primers, including 341F (5’-CCTACGGGNGGCWGCAG-3′), 805R (5’-GACTACHVGGGTATCTAATCC-3′), 515F (5’-GTGYCAGCMGCCGCGGTAA-3′), 806R (5’-GGACTACHVGGGTWTCTAAT-3′), F (5’-GTGCCAGCMGCCGCGG-3′) and R (5’-CCGTCAATTCMTTTRAGTTT-3′), are used to amplify the V3-V4 (464 bp), V4 (292 bp) and V4-V5 (392 bp) regions of 16S rDNA. The extracted DNA (25 ng per) was eluted in 50 μL of elution buffer (Nuclease-free TE Buffer) and stored at −80°C for subsequent PCR analysis, which was conducted by LC-Bio Technology Co., Ltd. (Hangzhou, Zhejiang, China). For PCR amplification, primers were tagged at the 5′ end with sample-specific barcodes and universal sequencing primers. Each 25 μL PCR reaction contained 25 ng of template DNA, 12.5 μL of PCR premix, 2.5 μL of each primer, and PCR-grade water to adjust the volume. The amplification conditions were as follows: initial denaturation at 98°C for 30 s, followed by 32 cycles of denaturation at 98°C for 10 s, annealing at 54°C for 30 s, and extension at 72°C for 45 s, with a final extension at 72°C for 10 min. PCR products were verified by 2% agarose gel electrophoresis. The electrophoresis buffer is 1 × TBE, with the voltage set at 98 V and a duration of 30 min. To eliminate false-positive results, ultrapure water was used as a negative control during DNA extraction. Purification of PCR products was performed using AMPure XT beads (Beckman Coulter Genomics, United States), and DNA quantification was carried out using the Qubit Fluorometer (Invitrogen, United States). Library size and concentration were assessed with the Agilent 2100 Bioanalyzer (Agilent, United States) and the Illumina Library Quantification Kit (Kapa Biosciences, United States). Sequencing was conducted on the NovaSeq PE250 platform (Logue et al., 2016).

#### 2.2.2 16S rRNA data analysis

Samples were sequenced on the Illumina NovaSeq platform according to the manufacturer’s protocol (provided by LC-Bio). Paired-end reads were assigned to individual samples based on barcodes and merged using FLASH. To ensure high-quality sequencing data, raw reads were filtered with fqtrim (v0.94), and chimeric sequences were removed using Vsearch (v2.3.4). DADA2 was applied for dereplication, generating feature tables and feature sequences. To analyze microbial diversity, samples were normalized by random subsampling for *α*-diversity and *β*-diversity calculations. Feature abundance was normalized using the SILVA classifier (v132). α-Diversity was assessed using the Chao1, Observed OTUS, Goods Coverage and Shannon indices to evaluate species richness and diversity (calculated with QIIME2). β-Diversity was also computed using Principal component analysis (PCA). Feature sequences were aligned and annotated against the SILVA database using BLAST, and additional graphs were generated using R (v3.5.2) (Takai and Horikoshi, 2000).

### 2.3 Metabolomics analysis

#### 2.3.1 Metabolite extraction

The collected samples were thawed on ice, and metabolites were extracted with 50% methanolBuffer. Briefly, 20 μL of sample was extracted with 120 μL of precooled 50% methanol, vortexed for 1 min, and incubated at room temperature for 10 min; the extraction mixture was then storedovernight at −20°C. After centrifugation at 4,000 g for 20 min, the supernatants were transferred intonew 96-well plates. The samples were stored at −80°C prior to the LC–MS analysis. In addition, pooledQC samples were also prepared by combining 10 μL of each extraction mixture.

#### 2.3.2 LC–MS conditions for metabolomics analysis

Samples were analyzed using a SCIEX TripleTOF 5,600 Plus mass spectrometer (United Kingdom) in both positive and negative ion modes. Chromatographic separation was conducted on a Waters ACQUITY UPLC T3 column (100 mm × 2.1 mm, 1.8 μm). The mobile phase consisted of 0.1% formic acid in water (A) and 0.1% formic acid in acetonitrile (B). The gradient elution program was as follows: 0–0.5 min, 5% B; 0.5–7 min, 5–100% B; 7–8 min, 100% B; 8–8.1 min, 100–5% B; and 8.1–10 min, 5% B. The flow rate was set at 0.4 mL/min, with the column temperature maintained at 35°C. Mass spectrometry parameters were configured as follows: curtain gas at 30 PSI, ion source gas 1 and gas 2 at 60 PSI, interface heating temperature at 650°C, and ion spray voltage set to 5 kV for positive ion mode and −4.5 kV for negative ion mode. Mass calibration was performed every 20 samples, and quality control (QC) samples were included every 10 samples to ensure analytical stability (Patti, 2011).

#### 2.3.3 Metabolomics data analysis

LC–MS data were preprocessed using XCMS software, incorporating the XCMS, CAMERA, and metaX toolboxes in R. Ions were identified based on retention time and m/z, generating a three-dimensional matrix containing peak indices, sample names, and ion intensities. Metabolites were annotated using the KEGG and HMDB databases with a mass error tolerance of 10 ppm. The preprocessing workflow included the removal of features with low detection rates, imputation of missing values, identification of outliers and batch effects using principal component analysis (PCA), and correction of signal drift using quality control (QC) samples. To ensure data reliability, the relative standard deviation (RSD) of metabolite features in QC samples was calculated, and features with RSD > 30% were excluded. Data normalization was performed using the probabilistic quotient normalization (PQN) algorithm, followed by spline-based batch correction using QC samples. For statistical analysis, Student’s t-test was applied to determine *p*-values, and multiple testing correction was conducted using the false discovery rate (FDR) method (Benjamini–Hochberg). Additionally, supervised partial least squares-discriminant analysis (PLS-DA) was performed using metaX, and significant features were identified based on a variable importance in projection (VIP) score > 1.0 (Wishart, 2019).

### 2.4 Correlation data analysis

Significant differences between groups were analyzed using one-way analysis of variance (ANOVA) in SPSS 25.0 (Chicago, United States), with a significance threshold set at *p* < 0.05. To examine associations between microbial communities and metabolites, Spearman correlation analysis was conducted using genus-level microbial taxa and differentially abundant secondary metabolites. A scaled heatmap of the correlation matrix was then generated using default clustering methods to visually represent the correlation patterns (Huang et al., 2021).

## 3 Results

### 3.1 Rice flowering enhanced gut microbiota in yellow catfish under the RFFM

#### 3.1.1 Increased gut microbial diversity and richness after Rice flowering

**Figure 2:**
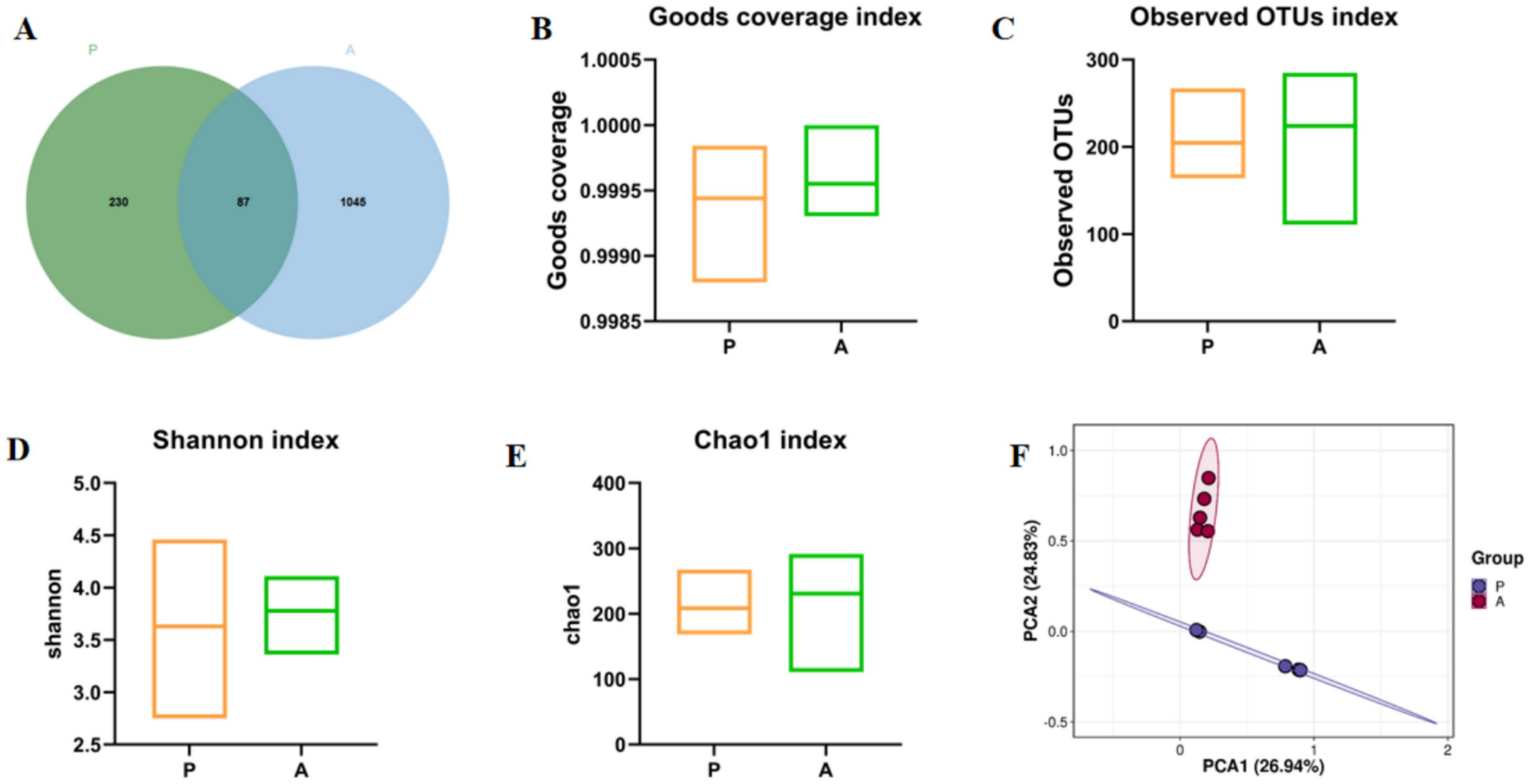
Venn diagram and *α*- and *β*-diversity analysis of gut microbiota. **(A)** OTU index; **(B)** Goods coverage index; **(C)** Observed OTUs index; **(D)** Shannon index; **(E)** Chao1 Index; **(F)** PCA Analysis. Group: P (pre-flowering), A (after-flowering).

Yellow catfish were introduced into the rice-fish symbiosis farming system and fed a compound diet. Intestinal contents were collected at both the Group P and Group A stages, and gut microbiota was analyzed via 16S rRNA sequencing. The results revealed a substantial increase in operational taxonomic units (OTUs) in Group A (1,045 OTUs) compared to Group P (230 OTUs), with 87 OTUs shared between the two groups (Figure 2A). Alpha diversity analysis further indicated significantly higher Shannon, Chao1, Observed_OTU, and Goods_Coverage indices in Group A compared to Group P (*p* < 0.05; Figures 2B–E). Principal Component Analysis (PCA) showed a clear separation between the two groups, as demonstrated by distinct confidence ellipses (Figure 2F). These findings suggest that rice flowering significantly enhances gut microbial diversity and richness in yellow catfish under the RFFM.

#### 3.1.2 Rice flowering reshapes dominant gut microbiota in yellow catfish

**Figure 3:**
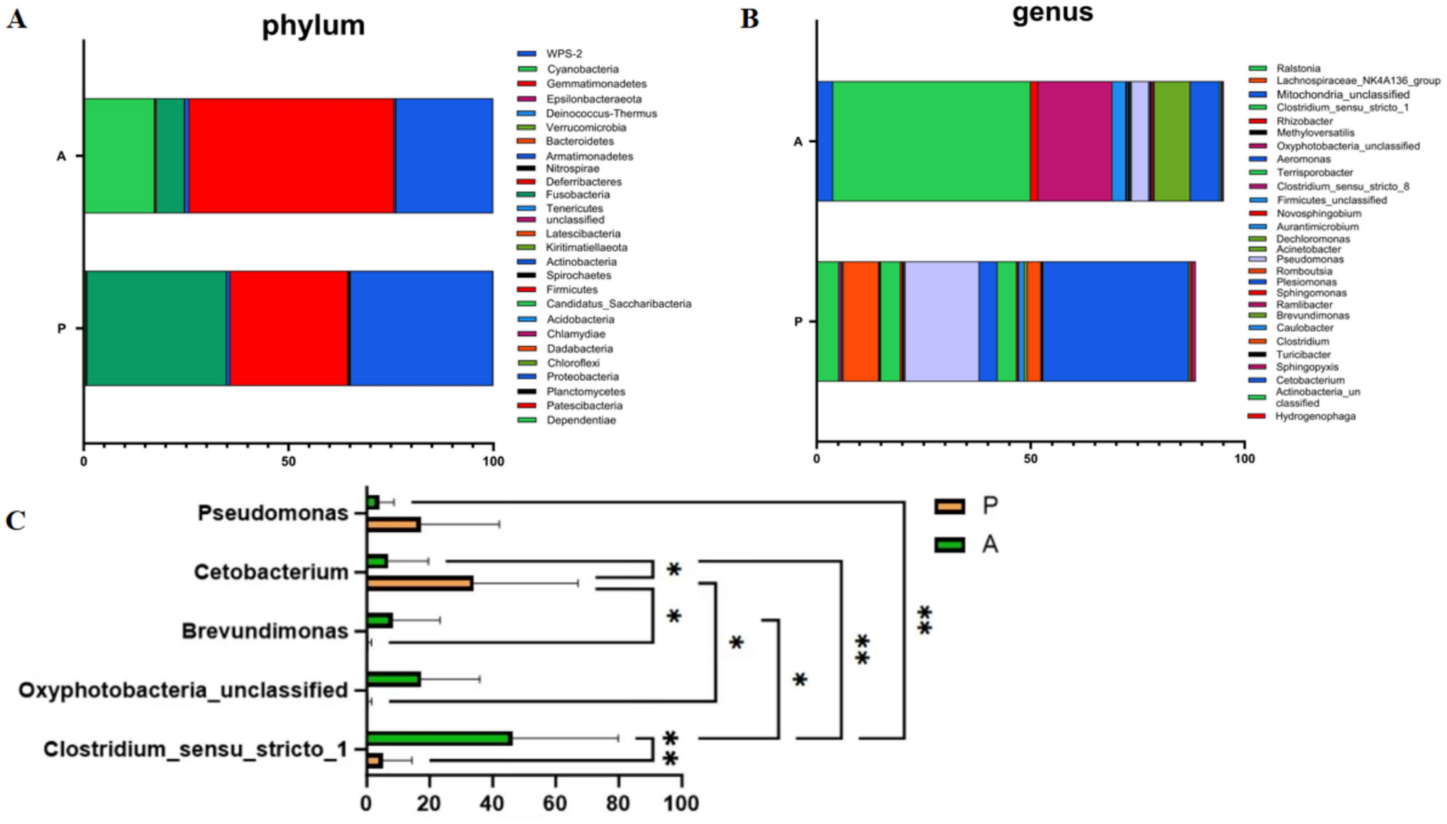
Composition and analysis of gut microbiota at phylum and genus levels. **(A)** Composition analysis of gut microbiota at the phylum level; **(B)** Composition analysis of gut microbiota at the genus level; **(C)** Differential microbial analysis at the genus level (*p < 0.05, **p < 0.01). Group: P (pre-flowering), A (after-flowering).

To assess changes in the dominant gut microbiota of yellow catfish between the pre-flowering (Group P) and after-flowering (Group A) phases under the rice-fish symbiosis farming model, we analyzed gut microbiota composition at both the phylum and genus levels. At the phylum level, the top 10 bacterial taxa in terms of relative abundance were *Firmicutes*, *Proteobacteria*, *Fusobacteria*, *Cyanobacteria*, *Actinobacteria*, *Bacteroidetes*, *Acidobacteria*, *Planctomycetes*, *Chloroflexi*, and *Dadabacteria* (Figure 3A). At the genus level, the most abundant taxa were *Clostridium_sensu_stricto_1*, *Cetobacterium*, *Pseudomonas*, *Oxyphotobacteria_unclassified*, *Brevundimonas*, *Romboutsia*, *Terrisporobacter*, *Plesiomonas*, *Ralstonia*, and *Mitochondria_unclassified* (Figure 3B). Notably, Firmicutes showed a higher relative abundance in Group A compared to Group P. Differential analysis revealed that *Pseudomonas* and *Cetobacterium* were significantly more abundant in Group P, whereas *Brevundimonas*, *Oxyphotobacteria_unclassified*, and *Clostridium_sensu_stricto_1* were significantly enriched in Group A (Figure 3C). These findings suggest that rice flowering in the RFFM significantly reshapes the dominant gut microbiota of yellow catfish. After flowering, the predominant gut microbial taxa consisted of *Brevundimonas*, *Oxyphotobacteria_unclassified*, and *Clostridium_sensu_stricto_1*.

### 3.2 Significant changes in hepatic metabolism of yellow catfish after rice flowering in the RFFM

#### 3.2.1 Rice flowering modified hepatic metabolic characteristics in yellow catfish

**Figure 4:**
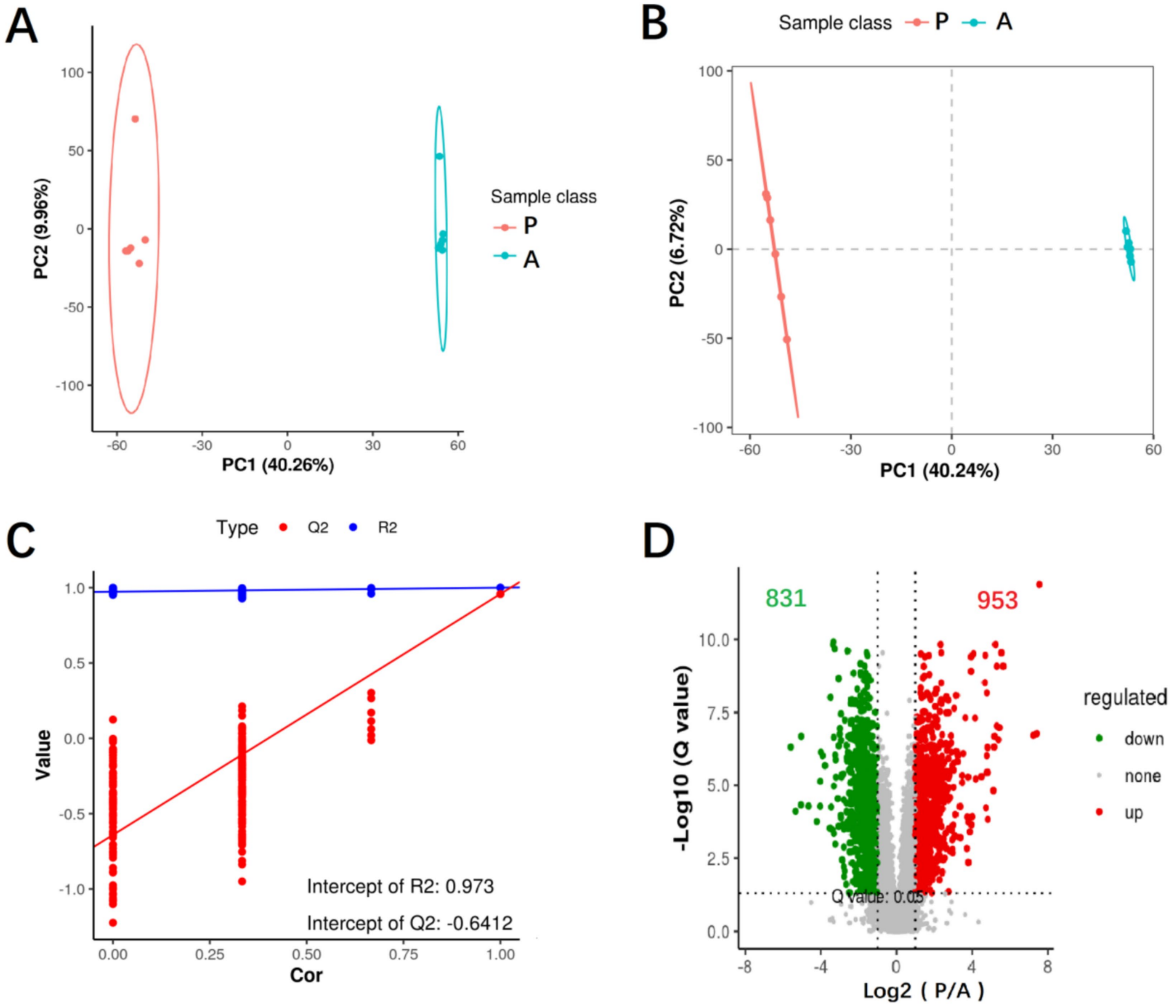
Analysis of differential hepatic metabolites. **(A)** PCA analysis of differential hepatic metabolites; **(B)** PLS-DA analysis of differential hepatic metabolites; **(C)** PLS-DA score plot of differential hepatic metabolites; **(D)** Volcano plot analysis of differential hepatic metabolites. Group: P (pre-flowering), A (after-flowering).

To assess metabolic changes between the pre-flowering (Group P) and after-flowering (Group A) phases, hepatic metabolic profiles were analyzed using untargeted metabolomics. Principal component analysis (PCA) demonstrated distinct clustering between the two groups (Figure 4A), while partial least squares-discriminant analysis (PLS-DA) further confirmed clear separation of confidence ellipses between Group A and Group P (Figure 4B). The PLS-DA model showed high reliability, with *R*^2^ = 0.973 and *Q*^2^ < 0, indicating a robust differential metabolite analysis (Figure 4C). Differential metabolites were identified based on the following criteria: fold change (FC) ≥ 2 or ≤ 0.5, variable importance in projection (VIP) > 1.0, and *p* < 0.05. Compared to Group A, Group P exhibited 953 upregulated and 831 downregulated metabolic ions (Figure 4D). These findings suggest that rice flowering in the RFFM induces significant alterations in the hepatic metabolic profile of yellow catfish.

#### 3.2.2 Rice flowering upregulates hepatic amino acid metabolism in yellow catfish

**Figure 5:**
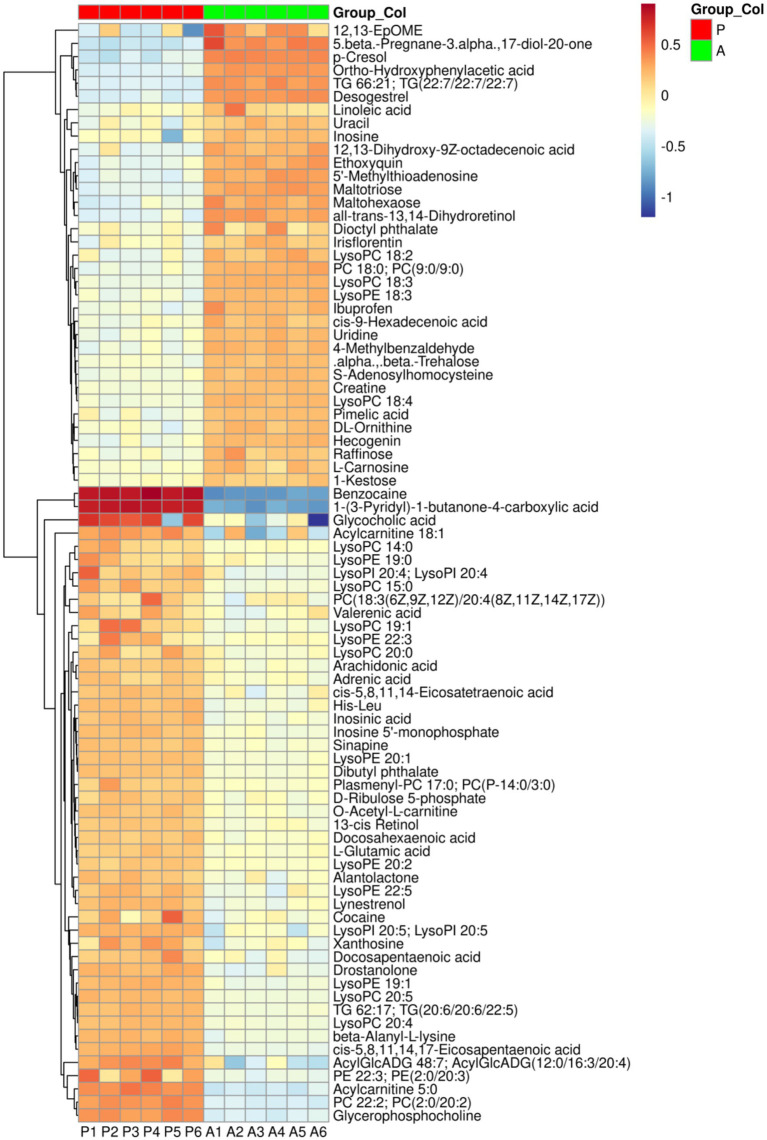
The Heatmap of differential metabolites between the two groups. Group: P (pre-flowering), A (after-flowering).

**Figure 6:**
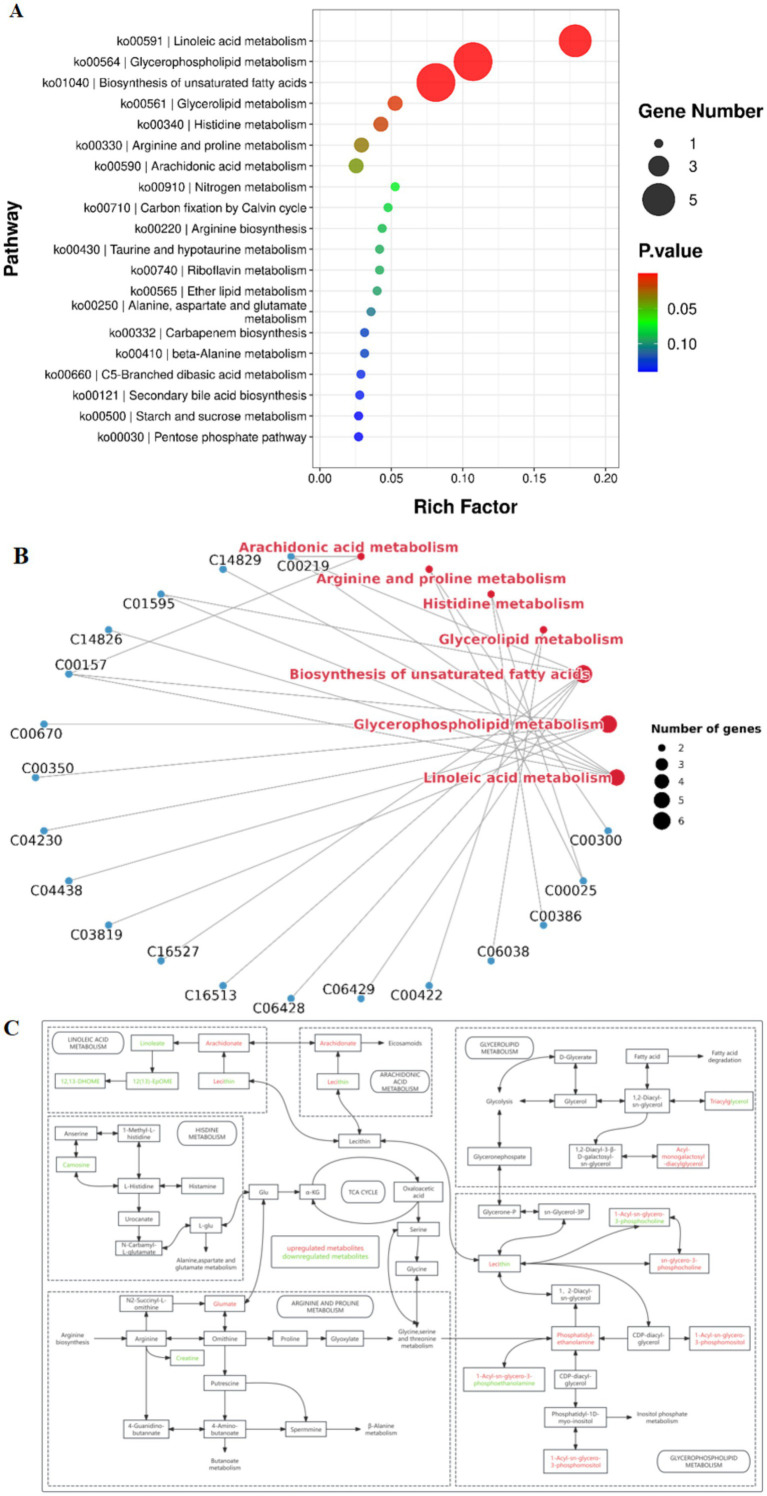
Pathway analysis of differential metabolites between the two groups. **(A)** KEGG pathway enrichment analysis; **(B)** Pathway network analysis; **(C)** Metabolic pathway interaction network.

Metabolic ions were annotated using the KEGG database, and a heatmap was generated to visualize differences in metabolite abundance between the two groups (Figure 5). Compared to the after-flowering group (Group A), the pre-flowering group (Group P) exhibited significant downregulation of metabolites, including uracil, inosine, uridine, creatine, maltotriose, maltohexaose, raffinose, linoleic acid, TG 66:21 [TG(22:7/22:7/22:7)], PC 18:0 [PC(9:0/9:0)], and LysoPC 18:3. Conversely, metabolites such as cis-5,8,11,14,17-eicosapentaenoic acid, docosapentaenoic acid, cis-5,8,11,14-eicosatetraenoic acid, arachidonic acid, docosahexaenoic acid, L-glutamic acid, His-Leu, LysoPC 19:1, and LysoPE 19:1 were significantly upregulated in Group A. To further explore the biological significance of these metabolic shifts, KEGG pathway enrichment analysis was conducted. The results revealed that the differential metabolites were primarily enriched in pathways related to linoleic acid metabolism, glycerophospholipid metabolism, biosynthesis of unsaturated fatty acids, histidine metabolism, and arginine and proline metabolism (Figure 6A). Pathway network analysis further demonstrated that key metabolites, including cis-5,8,11,14-eicosatetraenoic acid (C00219), linoleic acid (C01595), PC 22:2 (C00157), and L-glutamic acid (C00025), were involved in multiple metabolic pathways (Figure 6B). Further screening for shared metabolites across these pathways, we constructed a metabolic pathway correlation network (Figure 6C). The enriched metabolic pathways primarily revolved around two key metabolites, Glu and Lecithin, encompassing amino acid metabolism and lipid metabolism. In Arginine and proline metabolism, glutamate was upregulated while creatine was downregulated. In Histidine metabolism, the metabolite carnosine was downregulated. Arachidonate was upregulated in both Linoleic acid metabolism and Arachidonic acid metabolism, whereas linoleate, 12(13)-EpOME, and 12,13-DHOME were downregulated in Linoleic acid metabolism. In Glycerophospholipid metabolism, sn-glycero-3-phosphocholine, 1-acyl-glycero-3-phosphoinositol, and phosphatidyl-ethanolamine were upregulated. In glycerolipid metabolism, acyl-monogalactosyl-diacylglycerol was upregulated. These findings suggest that rice flowering in the RFFM downregulates hepatic lipid metabolism while promoting amino acid metabolism in yellow catfish. These metabolic adaptations likely play a crucial role in enhancing the fish’s ability to respond to environmental changes within the rice-fish ecosystem.

### 3.3 Gut dominant microbiota mediates hepatic metabolic changes induced by Rice flowering in yellow catfish

**Figure 7:**
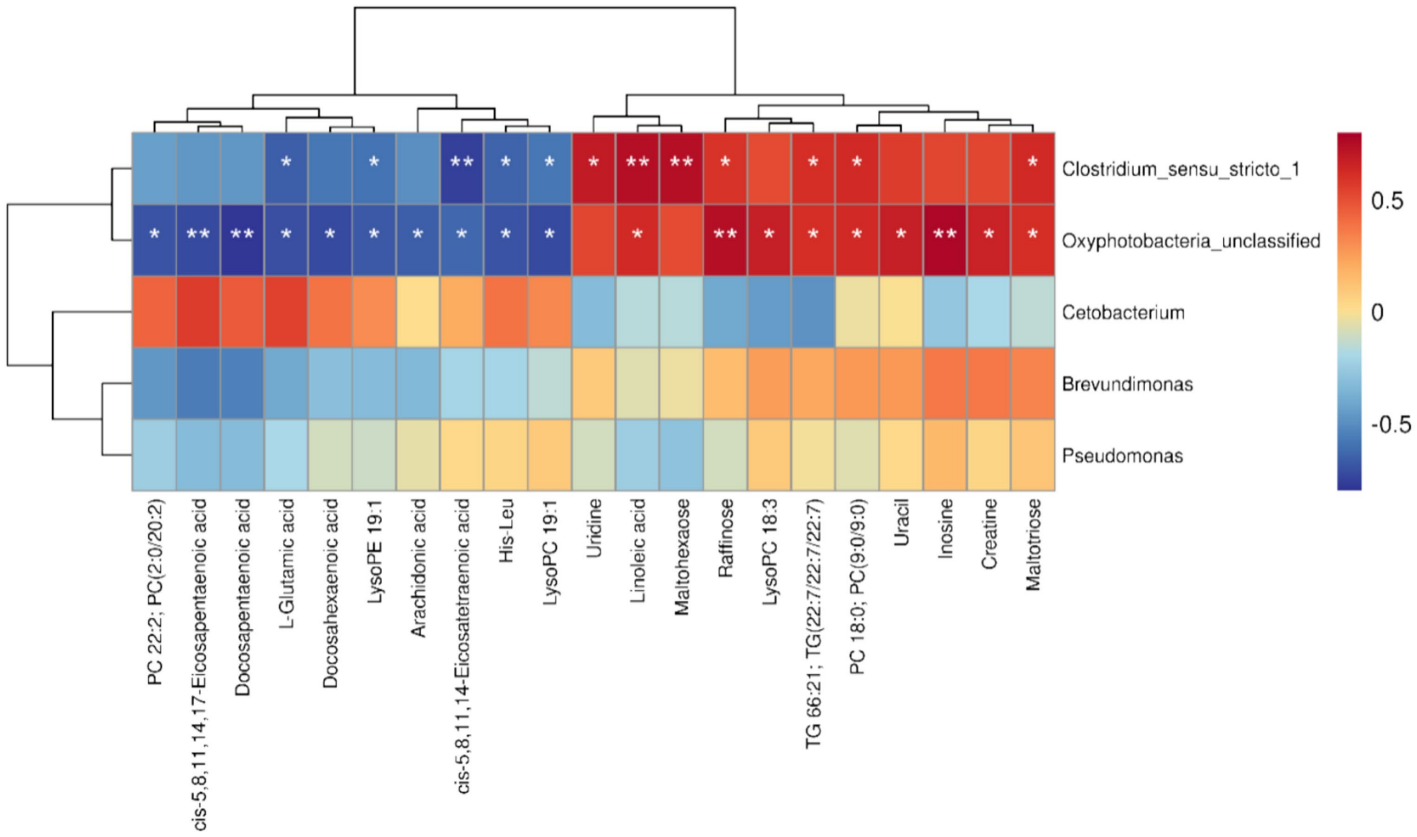
Spearman correlation analysis between differential microbial genera and differential metabolites (*p < 0.05, **p < 0.01).

To investigate the interactions between gut microbiota and hepatic metabolites in yellow catfish following rice flowering in the RFFM, we conducted a Spearman correlation analysis between differentially abundant microbial taxa and metabolites. The results revealed a significant positive correlation between *Clostridium_sensu_stricto_1* and several metabolites, including maltotriose (R = 0.64, *p* < 0.05), PC 18:0 [PC(9:0/9:0), *R* = 0.63, *p* < 0.05], TG 66:21 [TG(22:7/22:7/22:7), *R* = 0.61, *p* < 0.05], uridine (*R* = 0.70, *p* < 0.05), linoleic acid (*R* = 0.74, *p* < 0.01), and maltohexaose (*R* = 0.74, *p* < 0.01). In contrast, *Clostridium_sensu_stricto_1* exhibited a significant negative correlation with LysoPC 19:1 (*R* = −0.59, *p* < 0.05), His-Leu (*R* = −0.64, *p* < 0.05), LysoPE 19:1 (*R* = −0.60, *p* < 0.05), and L-glutamic acid (*R* = −0.71, *p* < 0.05). However, no significant correlations were observed between *Brevundimonas*, *Cetobacterium*, *Pseudomonas*, and the differentially abundant metabolites. These findings suggest that *Clostridium_sensu_stricto_1*, as a dominant gut microbiota member, plays a key role in modulating hepatic metabolic shifts in yellow catfish under the RFFM. This highlights the potential involvement of gut microbiota in metabolic adaptations triggered by environmental changes in the rice-fish ecosystem (Figure 7).

## 4 Discussion

The rice-fish symbiosis farming model (RFFM) has been shown to enhance gut microbial diversity and improve immunity in fish (Venegas et al., 2019; Zhu et al., 2024; Samaddar et al., 2025; Liu et al., 2024). Previous studies indicate that rice flowering influences gut microbiota composition and hepatic metabolism in carp (Tao et al., 2022). While probiotics play a crucial role in fish nutrition, immune regulation, and environmental adaptation, non-fish-derived probiotics often exhibit limited colonization efficiency and efficacy (Feng et al., 2025). This study aimed to explore the relationship between gut microbiota and hepatic metabolism in yellow catfish before and after rice flowering in the RFFM, with the objective of identifying host-derived probiotic candidates. Our findings indicate that rice flowering increases gut microbial diversity and richness, reshapes dominant microbial communities, downregulates hepatic lipid metabolism, and upregulates amino acid metabolism. Moreover, *Clostridium_sensu_stricto_1* appears to mediate hepatic metabolic changes in yellow catfish after flowering, contributing to their adaptation to environmental changes in the rice paddy ecosystem.

Microbial diversity and richness are essential for maintaining gut microbiota homeostasis (Chen-Liaw et al., 2025). In this study, the gut microbiota of yellow catfish exhibited significantly greater microbial abundance in the after-flowering group than in the pre-flowering group. The *α*-diversity indices were also higher in the after-flowering group, further supported by *β*-diversity analysis, which confirmed a distinct shift in microbial composition. These findings are in line with Jiang’s research on Gibel carp, which showed that rice flowering can enhance gut microbiota diversity in fish (Jiang et al., 2025). Among the dominant phyla, Firmicutes increased in abundance after flowering, likely due to the increased availability of natural prey in the rice paddy ecosystem (Nguyen et al., 2025). However, rice flowering also altered the composition of dominant gut microbiota in yellow catfish. Pseudomonas, a conditional pathogen known to cause fish mortality and negatively impact aquaculture sustainability (Li et al., 2020; Abou Elez et al., 2024), was significantly reduced after flowering. In contrast, Brevundimonas, a common genus in fish microbiota associated with improved growth and intestinal antioxidant activity (Feng et al., 2025), increased in abundance. Additionally, *Clostridium_sensu_stricto_1*, a Gram-positive anaerobe dominant in freshwater fish (Liang et al., 2024), plays a key role in gut health by producing short-chain fatty acids (SCFAs) through carbohydrate fermentation, which contributes to immune regulation (Srednicka et al., 2023). *Oxyphotobacteria_unclassified*, an oxygenic photosynthetic bacterium, may further support fish growth by maintaining dissolved oxygen levels under anaerobic conditions (Zhang et al., 2014). Collectively, these findings suggest that rice flowering in the RFFM reduces pathogenic bacteria while increasing beneficial gut microbes, thereby improving the gut microbiota composition of yellow catfish.

As the liver serves as the primary metabolic organ in fish, its metabolic activity is closely linked to nutrition and immune function (Sun et al., 2024). In this study, metabolomic analysis revealed a greater number of upregulated hepatic metabolites in the pre-flowering group compared to the after-flowering group, indicating that rice flowering accelerates hepatic metabolic processes in yellow catfish. Specifically, the levels of inosine and uridine increased after-flowering, both of which are known to enhance immune function (Yu and Li, 2025; Zhou et al., 2025). Furthermore, differential metabolite analysis revealed significant shifts in hepatic lipid and amino acid metabolism, with key metabolic pathways including linoleic acid metabolism, glycerophospholipid metabolism, biosynthesis of unsaturated fatty acids, histidine metabolism, and arginine and proline metabolism. After-flowering, L-glutamic acid levels decreased, while creatine levels increased, indicating a shift toward amino acid metabolism. In the amino acid metabolic pathway, proline is catalyzed by proline dehydrogenase (PRODH) or proline oxidase (POX) to generate ornithine, a precursor of arginine. Arginine is subsequently converted into creatine via the intermediate guanidinoacetic acid (GAA). Creatine plays a key role in inhibiting lipid synthesis while promoting fatty acid *β*-oxidation, thereby reducing lipid deposition in muscle tissue, maintaining muscle health, and improving metabolic status (Adeshina and Abdel-Tawwab, 2021). Studies have also shown that dietary creatine supplementation enhances water retention, texture, and flavor compounds in grass carp muscle (Gong et al., 2024).

Histidine metabolism is another essential pathway influenced by rice flowering. Histidine is deaminated by histidine ammonia-lyase to produce urocanic acid, which is subsequently converted into L-glutamic acid via urocanate hydratase. As a key intermediate, L-glutamic acid participates in multiple amino acid metabolic pathways and interconverts with L-aspartate through the *α*-ketoglutarate (α-KG) reaction. α-KG is a critical intermediate in the tricarboxylic acid (TCA) cycle, linking carbohydrate metabolism to energy production and degradation. Additionally, α-KG serves as a precursor for nucleotide biosynthesis, influencing nucleotide metabolism (Arnold and Finley, 2023). L-glutamic acid is also essential in the urea cycle, where it is deaminated by glutamate dehydrogenase (GDH) to produce ammonia, which is subsequently converted into urea for excretion, facilitating detoxification (Zhang et al., 2024).

Conversely, rice flowering downregulated lipid metabolism, as indicated by the decreased levels of cis-5,8,11,14-eicosatetraenoic acid and linoleic acid after-flowering. In lipid metabolism, lecithin-derived linoleic acid is elongated into longer-chain polyunsaturated fatty acids through elongase enzymes (Roy et al., 2024). Additionally, in the glycerophospholipid metabolic pathway, cytosolic phospholipase A2 (cPLA2) hydrolyzes membrane phospholipids, releasing arachidonic acid, a precursor for bioactive lipid mediators (Yang et al., 2024). The biosynthesis of arachidonic acid relies on desaturases such as fatty acid desaturase 1 (FADS1), which functions as a rate-limiting enzyme in this pathway (Xu et al., 2023). Collectively, these metabolic shifts suggest that yellow catfish adapt to the RFFM by downregulating lipid metabolism while upregulating amino acid metabolism, optimizing energy utilization in response to environmental changes.

*Clostridium_sensu_stricto_1* plays a pivotal role in host lipid metabolism through multiple pathways. This genus is known to produce SCFAs, particularly butyrate, which strengthens intestinal barrier integrity, reduces inflammation, and indirectly influences lipid metabolism (Bourdeau-Julien et al., 2023). Butyrate also modulates lipid metabolism and energy homeostasis by regulating endocannabinoid levels, including anandamide and palmitoyl ethanolamide, both of which are key regulators of inflammation, muscle strength, and energy balance (Vijay et al., 2021). Since arachidonic acid is a precursor to endocannabinoids, and linoleic acid serves as a precursor for arachidonic acid, *Clostridium_sensu_stricto_1* may influence lipid metabolism via the endocannabinoid system. Previous studies have also shown that *Clostridium_sensu_stricto_1* is associated with the enrichment of lysophospholipids and phosphatidylcholine metabolites in the glycerophospholipid pathway (Cai et al., 2024). Additionally, triglycerides such as TG 66:21 and TG (22:7/22:7/22:7), which participate in multiple lipid metabolic pathways, were significantly correlated with *Clostridium_sensu_stricto_1*. Our findings indicate a strong positive correlation between *Clostridium_sensu_stricto_1* and the levels of linoleic acid, TG 66:21, and TG (22:7/22:7/22:7), suggesting that this bacterial species plays a role in metabolic regulation under the RFFM.

Overall, our study demonstrates that rice flowering in the RFFM significantly reshapes the gut microbiota of yellow catfish, with *Clostridium_sensu_stricto_1* emerging as a key microbial mediator of hepatic metabolic changes. By enhancing metabolism and immune function, this bacterial species may serve as a potential probiotic for yellow catfish. These findings provide new insights into the development of host-derived probiotics for aquaculture.

## 5 Conclusion

In the rice-fish symbiosis farming model, the gut microbiota of yellow catfish improved significantly after rice flowering. The dominant bacterial genera in the gut microbiota after-flowering were *Brevundimonas*, *Oxyphotobacteria_unclassified*, and *Clostridium_sensu_stricto_1*. Hepatic metabolism also underwent substantial changes, with lipid metabolism downregulated and amino acid metabolism upregulated, likely as an adaptive response to environmental shifts in the rice paddy ecosystem. Notably, *Clostridium_sensu_stricto_1* played a key role in mediating hepatic metabolic adjustments, highlighting its potential influence on metabolic regulation in yellow catfish under the RFFM.

## Data Availability

The original contributions presented in the study are publicly available. This data can be found here: https://ngdc.cncb.ac.cn/bioproject/browse/PRJCA043453.
