# Supplementary material for: Changes of intestinal microbiota and liver metabolomics in yellow catfish (Pelteobagrus fulvidraco) before and after rice flowering in rice-fish symbiosis farmed mode
Source: Front Microbiol. 2025 Aug 6;16:1617168. doi: 10.3389/fmicb.2025.1617168 (PMC12364907; doi:10.3389/fmicb.2025.1617168)
Supplement: Supplementary file 1 [file Data_Sheet_1.pdf]

KEGG entry	compound name
C00129	Isopentenyl diphosphate
C14829	12,13-DHOME
C01595	Linoleate
C14826	12(13)-EpOME
C00157	Phosphatidylcholine
C00670	sn-Glycero-3-phosphocholine
C00350	Phosphatidylethanolamine
C04230	1-Acyl-sn-glycero-3-phosphocholine
C00438	N-Carbamoyl-L-aspartate
C03819	1-Acylglycerophosphoinositol
C16527	Adrenic acid
C16513	(7Z,10Z,13Z,16Z,19Z)-Docosapentaenoic acid
C06428	(5Z,8Z,11Z,14Z,17Z)-Icosapentaenoic acid
C06429	(4Z,7Z,10Z,13Z,16Z,19Z)-Docosahexaenoic acid
C00422	Triacylglycerol
C06038	Acyl1-monogalactosyl-diacylglycerol
C00386	Carnosine
C00025	L-Glutamate
C00300	Creatine
